# Role of CK2 inhibitor CX-4945 in anti-cancer combination therapy – potential clinical relevance

**DOI:** 10.1007/s13402-020-00566-w

**Published:** 2020-10-14

**Authors:** Claudio D’Amore, Christian Borgo, Stefania Sarno, Mauro Salvi

**Affiliations:** Department of Biomedical Sciences, University of Padova, Padova, Italy

**Keywords:** CX-4945, CK2, CK2 inhibition, Cancer, Leukaemia, Cancer therapy, Combination therapy

## Abstract

**Background:**

Protein kinase CK2 inhibition has long been considered as an attractive anti-cancer strategy based on the following considerations: CK2 is a pro-survival kinase, it is frequently over-expressed in human tumours and its over-expression correlates with a worse prognosis. Preclinical evidence strongly supports the feasibility of this target and, although dozens of CK2 inhibitors have been described in the literature so far, CX-4945 (silmitasertib) was the first that entered into clinical trials for the treatment of both human haematological and solid tumours. However, kinase inhibitor monotherapies turned out to be effective only in a limited number of malignancies, probably due to the multifaceted causes that underlie them, supporting the emerging view that multi-targeted approaches to treat human tumours could be more effective.

**Conclusions:**

In this review, we will address combined anti-cancer therapeutic strategies described so far which involve the use of CX-4945. Data from preclinical studies clearly show the ability of CX-4945 to synergistically cooperate with different classes of anti-neoplastic agents, thereby contributing to an orchestrated anti-tumour action against multiple targets. Overall, these promising outcomes support the translation of CX-4945 combined therapies into clinical anti-cancer applications.

## Introduction

It is widely acknowledged that the Ser/Thr protein kinase CK2, a tetrameric holoenzyme composed of two catalytic α (or α’) and a dimer of regulatory *β* subunits, is involved in the regulation of a plethora of biological processes and basal cellular functions, including growth, proliferation and differentiation, transcription and translation, glucose uptake, adhesion and migration [1–7]. This pleiotropy relays on the fact that CK2 is a ubiquitously expressed and constitutively active protein kinase, with hundreds of substrates widely distributed in most of the subcellular compartments [8, 9]. Indeed, different from most eukaryotic kinases, which are transiently activated in response to specific stimuli, CK2 is permanently in an active conformation. Not surprising according to its pro-survival and anti-apoptotic functions, CK2 inhibition is considered a promising therapeutic approach to treat different human tumours [10, 11]. To understand the relevance of inhibiting CK2 to treat cancer, two important aspects should be accounted for: 1) CK2 mRNA and/or protein are over-expressed in many malignancies, both haematological and solid and, usually, the increased expression of the kinase is related to a worse prognosis [12–15]. Nevertheless, CK2 should not be considered as a “true” oncogene, since its over-expression by itself is generally not sufficient to promote cancer development, but it makes the environment more suitable for cancer progression. This phenomenon is known as “non-oncogene addiction” [16]. 2) Cancer cells are more sensitive to CK2 inhibition compared to their healthy counterparts [16]. This aspect is extremely important as some concerns have been raised about the feasibility to inhibit a kinase that is involved in so many important biological processes.

**Fig. 1:**
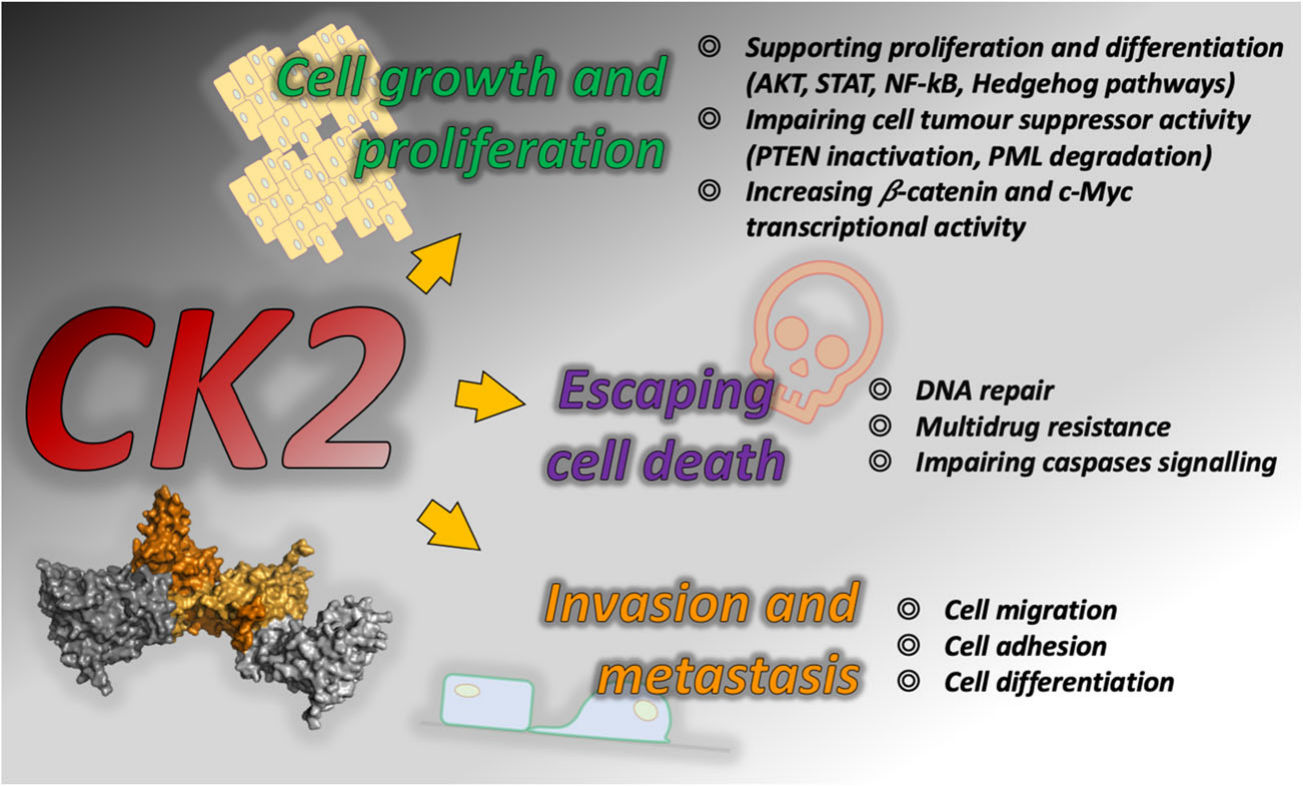
Schematic representation of the main cancer hallmarks in which the protein kinase CK2 has been implicated

CK2 takes part in some of the most important pathways that are relevant in cancer progression (Fig. 1), including the Hedgehog and Wnt, JAK/STAT, NF-κB, PTEN/PI3K/AKT and p53 pathways [17]. In particular, CK2 promotes tumorigenesis by regulating the activity of several oncogenes, i.e., its increased kinase activity has been related to an increased transcriptional activity of c-Myc and *β*-catenin, both of which are CK2 substrates. CK2-mediated phosphorylation protects them from degradation, thus boosting the expression of genes mainly involved in promoting cell proliferation and transformation [18]. In addition, several data have shown that CK2 may strongly impair tumour suppressor activities by inducing the phosphorylation of both PML and PTEN, resulting in the proteasomal degradation of the first and the stabilization of the latter in an inactive state [19–21]. Through the phosphorylation of AKT1 at Ser129, CK2 may also intervene in the pro-survival PI3K/AKT pathway contributing to perpetuate cell survival signalling [22]. In addition, a role of CK2 in regulating cell cycle progression at its different phases has been described [23]. Besides, it has been proposed that CK2 may impair caspase activation, i.e., due to similarity between the kinase consensus sequence and the caspase recognition motif, CK2-mediated phosphorylation of several caspase substrates could protect them from cleavage [24]. Finally, a role of CK2 in multidrug resistance has recently been reviewed, highlighting its involvement in promoting DNA repair and drug efflux, as well as in sustaining diverse signalling pathways to escape cell death [25].

Dozens of CK2 inhibitors have been described so far, which can be ranked into ATP-competitive and allosteric inhibitors. The first group comprises several small molecule inhibitors such as TBB, DMAT, IQA, CX-4945 and its derivative CX-5011, emodin, quercetin and ellagic acid, among others [26]. More recently, another family of 2-aminothiazole-derived compounds has been identified comprising non-ATP-competitive CK2 inhibitors which, by inducing the stabilization of the protein in an inactive form, hamper its kinase activity [27]. In addition, alternative inhibitory approaches have been attempted using synthetic peptides aiming at preventing holoenzyme assembly [28] or at counteracting CK2 substrate phosphorylation by targeting the conserved acidic phosphor-acceptor domain [29]. However, among all the CK2 inhibitors that have been identified so far, only a few had the pharmacological properties to overcome preclinical experimentation stages for cancer treatment. Among these, CX-4945 (5-(3-chlorophenylamino)benzo [c][2,6]naphthyridine-8-carboxylic acid), also known as Silmitasertib, is among the most promising ones, being effective both in vitro in cells and in vivo in animals models and being endowed with a suitable pharmacokinetic profile (long half-life, oral bioavailability, lack of toxicity) [30, 31], allowing to test its anti-tumour activity also in humans. Developed by Cylene Pharmaceuticals in 2011, CX-4945 is the first orally available small molecule CK2 inhibitor that acts as a potent ATP competitor, with an estimated K_i_ of 0.38 nM and an IC_50_ lesser than 3 nM [32, 33]. Moreover, this molecule has an excellent profile of selectivity: testing CX-4945 at 500 nM against a panel of 235 kinases (i.e., almost 50% of the known kinome) revealed that this compound is able to affect the activity of only 49 kinases to an extent greater than 50% [32].

**Fig. 2:**
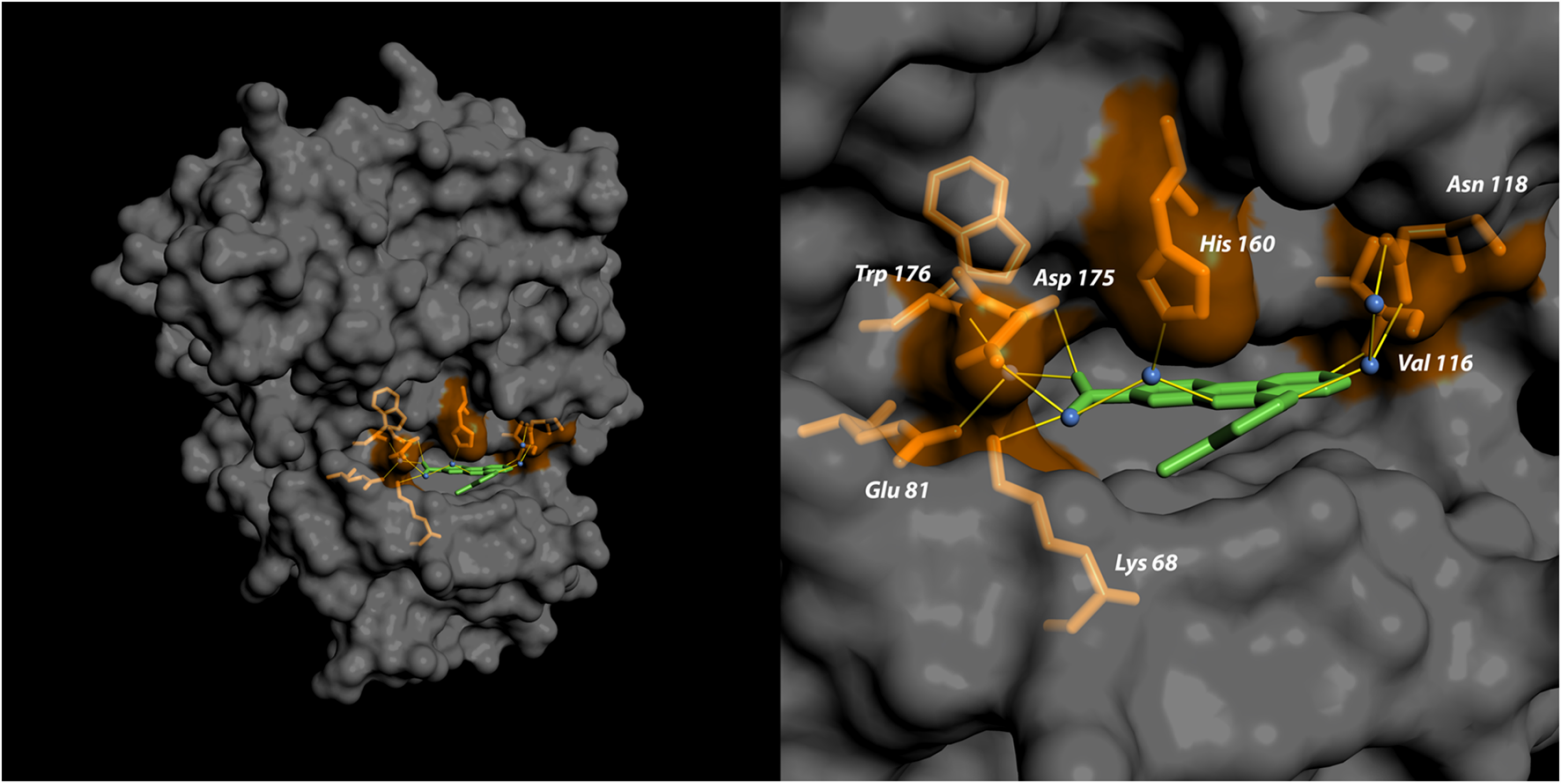
Crystal structure of the human CK2 catalytic α subunit (dark grey) in complex with CX-4945 (green); PDB code: 3PE1. In orange the amino acid residues involved in binding with the inhibitor are shown. The figure was generated using pyMOL software (https://pymol.org)

The crystal structure of human CK2α in complex with CX-4945 has for the first time been determined in 2011 [32, 34]. Consistent with its ATP-competitive mode of action, it showed a compound ability to interact with the ATP cavity of both CK2 α and α’, thereby establishing multiple hydrophobic interactions with the binding pocket. Additional analysis of the crystal structure revealed two direct interactions between the kinase and its inhibitor: the first one, between the CX-4945 pyridine group with a Val116 residue of the CK2 hinge region, and the second amongst the carboxylate group of CX-4945 and Lys68 of the CK2 *β*3 strand (Fig. 2). Additional contacts, involving the CX-4945 carboxylate group, are mediated by water molecules, which bridge interactions with Glu81, Asn118, His160, Asp175 and Trp176 residues [32, 34, 35].

When administered in cells, CX-4945 has been found to show an extensive anti-proliferative activity, i.e., to promote cell cycle arrest, and to induce caspase activity and apoptosis in various cancer cell lines [10, 11, 16]. In addition, CX-4945 has been found to strongly inhibit cell migration and differentiation [3]. These effects are strictly related to a rapid inhibition of CK2, since the reduction in phosphorylation of the main substrates of the kinase occurs within a few hours after CX-4945 administration [3, 33, 36]. Data obtained through a recent phospho-proteomic analysis performed in mitotic HeLa cells confirmed that CK2 is the main kinase target of CX-4945. Sequences of the major affected phospho-peptides were indeed found to be strongly acidophilic and enriched in aspartic and glutamic acid in position +1 and + 3 [37], thus suggesting that their reduced phosphorylation could be ascribed to CK2 inhibition [8, 38].

## CX-4945 in anti-tumour combination therapies

The concept of cancer addiction to CK2 implies that it is conceivable that not mere CK2 inhibition, but synergic actions of CK2 inhibitors with other chemotherapeutics may be more efficacious to treat tumours. Also, due to the multifactorial origin of cancer, the simultaneous targeting of multiple components is considered to be more promising [64, 65]. Several studies have documented the usefulness of exploiting CX-4945 in combination with different therapeutic drugs, and the ability of CK2 inhibitors to synergistically act with most of them. In this review, we will focus on combined anti-cancer therapeutic strategies involving the use of CX-4945 (Tables 1, 2 and 3).

**Table 1:** List of studies describing the use of CX-4945 in combination therapy for the treatment of haematological cancers. CI: Combination Index (< 0.5: strong synergism; 0.51–0.99: modest synergism; > 1–1,2: additivity)

Tumour	Cells used	Combined inhibitor	Target of the combined inhibitor	C.I.	Combined effect/s in vitro	Combined effect/s in vivo	Ref
Acute Lymphoblastic Leukaemia (ALL)	MOLT-4; JURKAT; CEM-R; KOPN-8; RS4,11; NALM-6 Primary ALL cells	Bortezomib	26S proteasome	0.1–0.9	• Inhibition of cell proliferation • Induction of apoptosis	–	[39]
Acute Lymphoblastic Leukaemia (ALL)	CCRF-CEM	Methotrexate	DHFR, TYMS, GARFT, AICART (Enzymes responsible for nucleotide synthesis)	0.44–0.75	• Inhibition of cell proliferation • Inhibition of cell cycle progression • Induction of apoptosis	–	[40]
Acute Lymphoblastic Leukaemia (ALL)	CEM	Vinblastine Doxorubicin	Tubulin DNA, DNA Topoisomerase II	0.7 -	• Inhibition of cell proliferation • Increased doxorubicin internalization	–	[41]
B- Acute Lymphoblastic Leukaemia (B-ALL)	SEM; RS4,11; NALM-6	Decitabine	DNA DNA methyltransferase	N.D.	• Reduced metabolic activity	• Reduced cell proliferation and dissemination	[42]
B- Acute Lymphoblastic Leukaemia (B-ALL)	NALM-6; 697; N6/ADR Primary B-ALL cells	Doxorubicin	DNA DNA Topoisomerase II	0.2–1.2	• Inhibition of cell viability • Induction of apoptosis • Overcoming doxorubicin resistance	• Reduced cell dissemination • Increased mice survival rate	[43]
T- Acute Lymphoblastic Leukaemia (T-ALL)	ALL-SIL; JURKAT; RPMI-8402; MOLT-3	JQ1	BRD4	0.16–0.75	• Inhibition of cell proliferation • Induction of apoptosis	–	[44]
Multiple Myeloma and Mantle Cell Lymphoma	INA-6; Jeko-1; Rec-1;U-266	Bortezomib	26S proteasome	0.01–0.84	• Inhibition of cell proliferation • Induction of apoptosis • Reduction of ATP production	–	[45]
Chronic Lymphocytic Leukaemia (CLL)	Primary CLL cells	GS-1101 Ibrutinib Fludarabine	PI3Kẟ BTK DNA polymerase α, Ribonucleotide reductase, DNA primase	0.46 0.56 0.3	• Inhibition of cell proliferation • Reduction of cell viability	–	[46]
Chronic Lymphocytic Leukaemia (CLL)	MO1043; MEC1; JVM3 Primary CLL cells	Fludarabine	DNA polymerase α, Ribonucleotide reductase, DNA primase	0.78–1.09 0.3–0.51	• Inhibition of cell proliferation • Overcoming fludarabine resistance	• Inhibition of tumour growth	[47]
Acute Myeloid Leukaemia (AML)	ML-2	Daunorubicin	DNA DNA Topoisomerase	0.86	• Inhibition of cell proliferation • Induction of apoptosis	–	[48]
Chronic Myeloid Leukaemia (CML)	LAMA-84; K562; KCL-22	Imatinib	BCR-ABL	0.57–0.87	• Induction of apoptosis • Overcoming Imatinib resistance	–	[49]

**Table 2:** List of studies describing the use of CX-4945 in combination therapy for the treatment of solid tumours. CI: Combination Index (< 0.5: strong synergism; 0.51–0.99: modest synergism; > 1–1,2: additivity)

Tumour	Cells used	Combined inhibitor	Target of the combined inhibitor	C.I.	Combined effect/s in vitro	Combined effect/s in vivo	Ref
Ovarian cancer	A1847; A2780; CP70; OVCAR5; SKOV3; UPN275	Dasatinib	BCR-ABL Src TKs	0.4–1.3	• Inhibition of cell proliferation • Inhibition of cell cycle progression • Induction of apoptosis	–	[50]
Ovarian cancer	A2780; SKOV3	Cisplatin Carboplatin Gemcitabine	DNA DNA DNA TYMS Ribonucleotide reductase	N.D.	• Inhibition of cell proliferation • Inhibition of cell cycle progression • Induction of apoptosis	• Inhibition of tumour growth • Increased apoptosis in xenografts	[51]
Cholangiocarcinoma	HuCCT1; EGI-1 Liv27	Gemcitabine Cisplatin	DNA TYMS Ribonucleotide reductase DNA	0.8–1.2	• Reduction of cell viability	• Inhibition of tumour growth • Reduction of xenografts proliferation rate • Increased apoptosis in xenografts	[52]
Cholangiocarcinoma	Primary CCA cells	MK2206 LY2157299	AKT TGFα receptor I	N.D.	• Inhibition of cell proliferation • Induction of apoptosis • Inhibition of cell migration	–	[53]
Gastric cancer	SNU-1	Paclitaxel	Tubulin	N.D.	• Inhibition of cell proliferation • Inhibition of cell cycle progression • Induction of apoptosis	• Inhibition of tumour growth • Reduction of xenografts proliferation rate • Reduction of pAKT(Ser473) and CK2 expression in xenografts	[54]
Medulloblastoma	Daoy; Med-1B; MB55;MB56	Temozolomide	DNA	0.02–0.61	• Inhibition of cell proliferation • Induction of apoptosis	–	[55]
Glioblastoma	GBM xenolines	Gefitinib	EGFR	N.D.	• Inhibition of cell proliferation	–	[56]
Glioblastoma	GL261	Temozolomide	DNA	N.D.	• Inhibition of cell proliferation	• Inhibition of tumour growth • Increased mice survival rate	[57]
Glioblastoma	SF767; U373; LN229	Temozolomide	DNA	N.D.	• Inhibition of cell proliferation	• Inhibition of tumour growth • Increased mice survival rate • Reduction of xenografts proliferation rate • Increased apoptosis in xenografts	[58]
Non-small cell lung carcinoma Squamous cell carcinoma	NCI-H2170 A431	Erlotinib	EGFR	N.D.	• Inhibition of cell proliferation • Induction of apoptosis	• Inhibition of tumour growth	[59]
Non-small cell lung carcinoma	A549; H460	AZD6244	MEK		• Inhibition of cell proliferation • Induction of apoptosis	–	[60]
Non-small cell lung carcinoma	A549; H460	Ionizing radiation	DNA	N.D.	• Inhibition of cell proliferation • Increased cell radio-sensitivity	–	[61]
Head and Neck squamous cell carcinomas	UM-SCC1; UM-SCC46	PD-0325901	MEK	N.D.	• Inhibition of cell proliferation	• Inhibition of tumour growth • Increased apoptosis in xenografts	[62]
	HeLa; HepG2	Heat shock	Heat shock proteins	N.D.	• Inhibition of cell proliferation • Increased cell sensitivity to hyperthermia	–	[63]

**Table 3:** Ongoing clinical trial using CX-4945

Identifier	Tumour	Combination therapy	The target of the combined inhibitor	Phase	Study completion	Locations
NCT03897036	Basal cell carcinoma	–	–	1	March 2021	USA
NCT02128282	Cholangiocarcinoma	Cisplatin Gemcitabine	DNA DNA TYMS Ribonucleotide reductase	1, 2	November 2021	USA Republic of Korea Taiwan
NCT03904862	Medulloblastoma	–	–	1, 2	May 2022	USA
NCT03571438	Kidney cancer	Ku 60,019	ATM	–	September 2024	France

### Haematological tumours

#### Acute lymphoblastic leukaemia

The efficacy of CX-4945 has been evaluated in a broad range of human haematological malignancies, including B- and T-cell acute lymphoblastic leukaemias (ALL), in which increases in both CK2 expression and activity have frequently been observed [15].

Bortezomib is a reversible inhibitor of the proteasome 26S subunit, whose targeting turned out to be a feasible strategy in several haematological malignancies [66]. Recently, it has been shown that CX-4945 enhanced bortezomib-induced apoptosis in a panel of both B- and T-ALL cell lines, as well as in primary ALL blasts from paediatric patients [39]. The combined treatment with bortezomib and CX-4945 prompted an endoplasmic reticulum stress-mediated cell death, favouring the accumulation of IRE-1α and CHOP, as well as an increased phosphorylation of ERK and EIF2α. Moreover, the combination of CX-4945/bortezomib induced an inhibition of the unfolded protein response (UPR) pathway and down-regulation of the anti-apoptotic mediator BCL-XL [39]. A strong synergic effect between bortezomib and CX-4945 was observed also in both multiple myeloma and mantle cell lymphoma cell lines, i.e., the combined treatment inhibited cell proliferation and impaired ATP production. In fact, in these tumour cells, the simultaneous inhibition of CK2 and the 26S proteasome caused a depolarization of the mitochondrial membrane, an increased expression of the pro-apoptotic proteins BAX and BAK, and decreased protein levels of anti-apoptotic Bcl-2 and Mcl-1, thus promoting apoptosis [45].

In ALL cells, a combined treatment with methotrexate and CX-4945 synergistically inhibited cell proliferation, blocked cell cycle progression in the G2/M phase and promoted the activation of caspases 3/7 [40]. The anti-tumour activity of methotrexate is brought about by inhibition of dihydrofolate reductase (DHFR), as well as of other enzymes involved in nucleotide synthesis. However, despite these promising results, it was found that a 48–72 h treatment with CX-4945 in CCRF-CEM T-ALL cells induced increased transcription of the *DHFR* gene. This side effect should be considered in the perspective of pharmacological therapy in humans, since it may promote the acquisition of methotrexate resistance.

More recently, promising preclinical results have been obtained with the combined administration of CX-4945 and decitabine, a chemical analogue of cytidine that, when incorporated into DNA, induces inhibition of DNA methyltransferase, thus causing DNA hypomethylation and reactivation of silenced tumour suppressor genes, leading to cell death [67]. Combined treatment with the CK2 inhibitor and the DNA-hypomethylating agent significantly decreased the metabolic activity of SEM and NALM-6 cells (two B-ALL cell lines), without affecting their proliferation rate. More interestingly, it was found that co-administration of decitabine and CX-4945 markedly reduced the proliferation and dissemination of SEM cells in a mouse xenograft model [42].

CX-4945 treatment has also been found to be effective in sensitizing multidrug-resistant CEM cells to the action of the anti-mitotic agent vinblastine, as well as in promoting doxorubicin accumulation [41]. Both vinblastine and doxorubicin are anti-neoplastic agents widely used in clinical practice for the treatment of various haematological and solid tumours through two different modes of action: the first one, by blocking microtubule assembly, induces cell cycle arrest in the M phase and prohibits cell division, whereas the latter is a DNA intercalating agent able to promote DNA strand breaks and to inhibit DNA topoisomerase II. The observations that CX-4945 enhances the effect of vinblastine and facilitates doxorubicin uptake within cells are useful in the context of overcoming the acquisition of chemoresistance by tumour cells, an adverse and common effect that typically arises during long-term chemotherapy. In this regard, the implication of the CK2/IKAROS axis in regulating the expression of *BCL2L1*, a gene that encodes the anti-apoptotic protein BCL-XL strictly related to the acquisition of chemoresistance in different blood-borne malignancies, has recently been shown [43]. IKAROS is a transcription factor endowed with tumour suppressor activity and its contribution to repressing the expression of several genes involved in cell cycle progression and the PI3K pathway is well known. Loss of IKAROS function has been associated with high-risk B-ALL and negative regulation of its activity by CK2 has been demonstrated [15, 43]. In their work, Song and colleagues showed that both CK2 silencing and CX-4945-mediated inhibition promoted IKAROS binding to the *BCL2L1* promoter, thereby significantly affecting its transcription [43]. Moreover, in B-ALL cell lines CX-4945 showed a strong synergy with doxorubicin and, most importantly, contributed to overcoming resistance to doxorubicin also in multidrug-resistant B-ALL cells. The efficacy of this combined treatment was also observed in a murine patient-derived xenograft model: co-administration of both drugs was more effective in increasing the survival rate, as well as in reducing leukaemia cell diffusion in spleen and bone marrow, compared to both CX-4945 and doxorubicin treatments alone [43].

A synergistic anti-cancer response (i.e., arrest of cell growth and promotion of apoptosis) has been observed in different T-ALL cell lines treated with CX-4945 in combination with JQ1, an inhibitor of the BET family member BRD4. Aberrant activation of the transcription factor c-Myc mediated by Notch1 is a crucial event in T-ALL initiation, and it was found that both CX-4945 and JQ1 impair this axis (the first hampering Notch-1 signalling, the latter reducing c-Myc expression), thereby contributing to reducing c-Myc transcriptional activity [44, 68].

#### Chronic lymphoblastic leukaemia

The feasibility of using CX-4945 in combined therapies has been explored also for the treatment of chronic lymphoblastic leukaemia (CLL). A first study was conducted in primary leukaemia cells from CLL patients to evaluate the efficacy of various combination therapies, in which CX-4945 administration was combined with three different drugs: fludarabine, a purine analogue inhibitor of DNA synthesis, GS-1101 (idelalisib), a phosphoinositide 3-kinase p110ẟ (PI3Kẟ) inhibitor, or ibrutinib, a potent and irreversible inhibitor of Burton’s tyrosine kinase (BTK) [46]. Both BTK and PI3Kẟ are important components of the B-cell receptor signalling pathway, which plays a pivotal role in the pathogenesis of CLL [69, 70]. In addition, idelalisib, ibrutinib and fludarabine have since long been approved for the treatment of CLL. The results of the work of Prins and colleagues showed that CX-4945 can synergistically enhance the anti-proliferative activity of all the three compounds tested [46]. The synergy between fludarabine and CX-4945 in CLL was further confirmed in the work of Martins and colleagues. Indeed, the authors showed that the combined treatment was effective both in vitro (inhibiting the survival in different CLL cell lines and primary CLL cells) and in vivo (delaying tumour growth in a mouse xenograft model using MO1043 cells). Interestingly, computation of the combination index revealed that the synergy was stronger in primary CLL cells than in their immortalized counterparts [47].

#### Acute myeloid leukaemia/chronic myeloid leukaemia

Acute myeloid leukaemia (AML) is characterized by an overproduction of immature white blood cells. It has been shown that CK2 is often over-expressed and hyper-activated in leukaemic blasts, thereby serving as an unfavourable prognostic marker in AML [71]. CX-4945 showed a moderate synergy when administered with the anti-neoplastic and DNA intercalating agent daunorubicin. More specifically, in acute myelomonocytic leukemia ML-2 cells, through inhibition of CK2, CX-4945 potentiated daunorubicin-induced apoptosis and suppression of cell proliferation [48].

Finally, the CK2 level was found to be markedly increased in imatinib-resistant LAMA-84 CML cells [49]. In this and other CML cell lines, it has been shown that CK2 co-localizes and interacts with the BCR-ABL fusion protein, an aberrantly activated tyrosine kinase resulting from the chromosomal translocation t(9;22)(q34;q11) and responsible for the onset of this malignancy. Although BCR-ABL is not a CK2 phosphorylation substrate, it was found that CX-4945 abrogates the CK2/BCR-ABL interaction and, most importantly, contributes to overcoming imatinib-resistance in different CML cell lines [49]. Notably, also CX-5011, a derivative of CX-4945, was found to induce apoptosis in CML cells and to promote a synergistic reduction in imatinib-resistant K562 and KCL22 cell viability when used in combination with imatinib and the MEK inhibitor U0126 [72].

### Solid tumours

It has also been reported that the combination of CX-4945 with different anti-neoplastic agents produced significant results in in vitro and in vivo models of solid tumours, including ovarian, gastrointestinal, brain, lung and other tumours.

#### Ovarian cancer

An analysis conducted in several epithelial ovarian cancer cell lines showed that the expression of CK2α is inversely correlated with sensitivity to dasatinib, a dual inhibitor of both BCR-ABL and Src family tyrosine kinases [50]. Dasatinib has been approved for the treatment of patients with CML and ALL. Several preclinical studies also suggested its potential application in ovarian cancer therapy, aiming at inhibiting Src kinases and their pro-tumour activities, since their contribution to the regulation of cell proliferation, adhesion and motility has been widely demonstrated [73]. Interestingly, in ovarian cancer cell lines co-administering CX-4945 and dasatinib synergistically reduced cell proliferation, and promoted caspase activation and apoptosis [50]. The feasibility of using CX-4945 to treat ovarian cancer was further demonstrated by combining the CK2 inhibitor with platinum-based drugs (i.e., cisplatin and carboplatin) or gemcitabine [51], an anti-neoplastic agent proposed as single-agent treatment for ovarian cancer [74, 75]. Gemcitabine is an anti-metabolite that mainly acts by inhibiting the activity of thymidylate synthetase, an enzyme involved in thymidine synthesis, thus leading to inhibition of DNA synthesis. At the same time, gemcitabine prevents the synthesis of deoxynucleotide triphosphates through inhibition of ribonucleotide reductase activity. In their work, Siddiqui et al. showed a synergy between CX-4945 and cisplatin, as well as between CX-4945 and gemcitabine, to inhibit cell cycle progression and proliferation, to promote DNA single- and double-strand breaks and activation of the caspase pathway. Furthermore, the aforementioned combined treatments were also found to be effective in vivo, showing a significant delay in tumour growth in mouse xenograft models [51].

#### Cancers of the gastrointestinal tract

The efficacy of combining CX-4945 with cisplatin and gemcitabine was further confirmed in a recent preclinical study conducted in both in vitro and in vivo cholangiocarcinoma models [52]. The favourable outcomes of this combined therapy are to be traced back to the inactivation of XRCC1 and MDC1 (two enzymes phosphorylated by CK2 and strictly involved in DNA repair) resulting from kinase inhibition mediated by CX-4945 [51, 52]. Again, CX-4945 appeared to be effective for the treatment of cholangiocarcinoma by eliciting inhibition of cell proliferation and migration, when co-administered with MK2206 (an AKT inhibitor) or LY2157299 (a TGFβR1 inhibitor) [53]. Finally, it should be mentioned that cholangiocarcinoma knockout cell lines for the α subunit of CK2 are significantly more sensitive to 5-fluorouracil and gemcitabine than their wild-type counterparts, two cytostatic drugs widely used for cholangiocarcinoma treatment [76], supporting the feasibility of a combinatory therapy with CX-4945 and these drugs.

Two independent groups have recently disclosed a key role of CK2 also in gastric cancer [12, 13]. It was shown that increased expression of the different CK2 subunits was associated with late-stages and a worse prognosis. In vitro studies conducted in SNU-1 gastric cancer cells revealed that combination of CX-4945 with paclitaxel significantly enhanced the anti-proliferative effects exerted by each agent alone. Moreover, co-administration of the CK2 inhibitor and the hyper-stabilizing microtubule agent evoked PARP cleavage and thus apoptosis induction. The anti-tumour efficacy of combining CX-4945 and paclitaxel was also demonstrated in SNU-1 xenografted mice. Indeed, although both monotherapies were effective in delaying xenograft tumour growth and proliferation rates, the greatest tumour reduction was observed in mice that received the combined treatment [54].

#### Brain tumours

In the last decade, several studies have been published showing the effectiveness of CX-4945 in combined therapies for the treatment of brain malignancies. In 2019, Nitta et al. reported that CK2 is involved in promoting medulloblastoma tumorigenesis, as well as the ability of CX-4945 to inhibit the proliferation of different medulloblastoma cell lines [55]. From a high-throughput analysis of 4000 FDA-approved compounds, the authors identified temozolomide as a molecule that could enhance the CX-4945 anti-cancer efficacy for the treatment of this neoplasm. In particular, they found that CX-4945 treatment in association with temozolomide strongly delayed cell growth and promoted apoptosis in vitro*.* Most importantly, computation of the combination index revealed a strong synergy between both drugs [55]. Temozolomide is a DNA alkylating agent used in clinical practice to treat patients affected with glioblastoma (GBM) and other brain cancers, including neuroblastoma and astrocytoma, and exerts its anti-tumour activity through methylation of the O^6^ position of guanosine, which in turn causes the formation of DNA single- and double-strand breaks. The main enzyme involved in repairing these specific DNA damages is O-6-methylguanine-DNA methyltransferase (MGMT), whose activity is positively regulated by the CK2/β-catenin pathway. Therefore, CK2 inhibition mediated by CX-4945 leads to inhibition of the MGMT DNA-repairing activity, thereby potentiating the efficacy of temozolomide [55, 77]. The relevance of targeting CK2 in GBM, one of the most aggressive brain tumours in adults, has been widely shown [56, 78, 79], and the efficacy of the CX-4945/temozolomide combination therapy has been proven also in this malignancy. Indeed, combining these two drugs reduced the proliferation of several GBM cell lines and effectively inhibited tumour growth in mouse xenograft models, significantly prolonging their survival compared to the single-drug treated ones [57, 58].

In the perspective of translating and scheduling CX-4945-based therapy in humans, special mention should be addressed to the work of Ferrer-Font et al. [57]. These authors showed that a six day-spaced metronomic administration of CX-4945 was more effective in inhibiting tumour growth in a mouse GBM model in comparison to both daily and every other day treatments, which both were almost ineffective. Besides, frequent CX-4945 administration also hampered the beneficial effect of temozolomide. Using C57BL/6 immunocompetent mice to better mimic a real clinical situation, the authors found a possible explanation for failure of the daily administration of CX-4945 in interference exerted by CK2 inhibition to the host immune system. Since CK2 inhibition has been associated with impairment of white blood cell differentiation, its constant inhibition may affect both immune cell recruitment and response [57].

Genetic *EGFR* alterations (mainly amplifications, but also rearrangements, point mutations and deletions) have been found in about 60% of GBM patients, leading to aberrant EGFR signalling and uncontrolled activation of its downstream pathways (MAPK, PI3K/AKT, JAK/STAT and others), that in turn promote cell and tumour growth, apoptosis resistance and angiogenesis. However, EGFR-targeted therapies have yielded poor results in patients with GBM [80, 81]. Interestingly, however, it has been observed that, when administered in conjunction with the EGFR inhibitor gefitinib, CX-4945 exerted a strong anti-proliferative effect on GBM xenolines in vitro [56].

#### Lung cancers

A further demonstration of the efficacy of combined therapies aimed at simultaneously inhibiting both CK2 and EGFR was obtained in both in vitro and in vivo models of lung cancer. Indeed, co-administration of CX-4945 and erlotinib was able to effectively attenuate the PI3K-AKT-mTOR pathway in both squamous cell carcinoma and non-small cell lung carcinoma (NSCLC) cell lines, leading to reduced activation of AKT and its substrates, thereby inhibiting proliferation and enhancing apoptosis. Importantly, the efficacy of the CX-4945/erlotinib combined therapy was confirmed in an in vivo mouse xenograft model, i.e., it significantly delayed tumour growth compared to the respective monotherapies [59]. Furthermore, a potential synergism between CX-4945 and selumetinib was also found to inhibit growth and induce PARP cleavage in NSCLC cell lines [60]. Selumetinib is a non-ATP competitive inhibitor of MEK 1/2, known components of the mitogen-activated protein kinase (MAPK) cascade, whose activation culminates in the phosphorylation and regulation of various transcription factors critically involved in cell survival, proliferation, differentiation and migration. Selumetinib has been evaluated in multiple phase II clinical trials for the treatment of various solid tumours [82]. However, unsatisfactory outcomes obtained in a recent international phase III clinical trial in which NSCLC patients were treated with selumetinib should be noted [83].

Recently, the feasibility of combining CX-4945-mediated CK2 inhibition with non-pharmacological anti-cancer treatments has been suggested. Indeed, pre-treating both A549 and H460 lung cancer cells with CX-4945, prior to X-ray exposure, increased their sensitivity to ionizing radiation, thereby significantly affecting the abilities of the cells to proliferate and form colonies [61].

#### Other solid tumours

As noted above for other malignancies, CK2 expression and activity have been found to be increased in head and neck squamous cell carcinoma (HNSCC) and to be associated with an aggressive form of the disease [62, 84]. The option that simultaneous inhibition of both CK2 and MEK may exert stronger anti-tumour outcomes has been explored in HNSCC cells by co-administration of CX-4945 and PD-0325901, a selective and non-ATP-competitive MEK inhibitor recently entered in clinical trials, whose anti-tumour efficacy has been shown in many preclinical studies in different human cancers [62, 85, 86, (https://clinicaltrials.gov/ct2/home)]. In these cells, MEK inhibition further potentiated the anti-proliferative effect mediated by CX-4945. Also, the combination of CX-4945 and PD-0325901 was more effective in delaying tumour growth in a mouse model compared to both monotherapies [62].

Previously, we revealed a relationship between CK2 and the small heat shock protein HSP27 [63], a protein with chaperone and anti-apoptotic activities considered to be an important player in thermo-tolerance [87, 88]. We showed that HSP27 regulation by CK2 is indirect and mediated by the ubiquitin ligase SMURF2. Moreover, we assessed the combined effects of CK2 inhibition and heat shock stress on proliferation in both HepG2 hepatocellular carcinoma cells and HeLa cervical cancer cells, revealing that CX-4945 pre-treatment strongly enhanced tumour cell thermo-sensitivity [63].

## Conclusions and perspectives

In the last twenty years, the idea to target kinases whose aberrant signalling is related to tumour initiation and/or progression, has been considered as one of the most promising approaches to treat cancer and, indeed kinase inhibitors currently constitute one of the largest families of anti-cancer drugs. Kinase inhibitor monotherapies, however, turned out to be effective only in a limited number of malignancies, usually driven by a single kinase, such as CML [89]. On the other hand, mounting evidence suggests that, due to the multifaceted causes that underlie neoplastic transformation, a multi-target approach should be preferred. Furthermore, the possibility that drug resistance may arise in tumour cells should always be taken in account, also for those tumours in which single treatment was initially effective.

It has been well-established that protein kinase CK2 plays a pivotal role in carcinogenesis due to its “horizontal” contribution in regulating several hierarchical transduction pathways involved in basal cellular functions, as well its considerable number of substrates. It is a commonly thought that CK2 should not be considered as a real oncogene, as its over-expression/up-regulation is generally not the prime cause of cancer development, but rather makes the cellular environment more suitable for cancer initiation and progression [10, 11, 16]. Therefore, the promising preclinical results obtained using CX-4945 as anti-tumour agent primarily rely on the unique/distinctive characteristics of its main target CK2, thereby allowing the drug to potentially affect multiple pro-survival pathways.

As we have already mentioned, CX-4945 has reached phases I and II clinical trials to test its anti-tumour activity (https://clinicaltrials.gov/ct2/home). The first approved clinical trials using CX-4945 as a single agent date back to 2010 (NCT01199718, NCT00891280). Interim reports from these trials showed that CX-4945 exhibited promising pharmacokinetic, pharmacodynamic and a safety profiles, and that over 15% of the treated patients showed disease stabilization for at least 6 months [90, 91]. But, to the best of our knowledge, the final results of these studies have not been published. Currently, CX-4945 is being used to treat patients with basal cell carcinoma (NCT03897036), cholangiocarcinoma (NCT02128282), kidney cancer (NCT03571438) and paediatric patients affected with medulloblastoma (NCT03904862). The aims of the ongoing trials are: 1) optimization of CX-4945 treatment by determination of the optimal and maximum tolerated doses; 2) assessment of drug pharmacokinetics and pharmacodynamics; 3) description of CX-4945-related adverse events; 4) demonstration of its anti-tumour activity. Interestingly, the multicentre cholangiocarcinoma study aims to translate the integration of CX-4945 into combined cisplatin/gemcitabine therapies, whose efficacy has been demonstrated in pre-clinical stages, to humans [52]. Notably, the U.S. Food and Drug Administration (FDA) has designated CX-4945 as an Orphan Drug for the treatment of cholangiocarcinoma in 2017 [92]. The results of these clinical trials will, however, not be available before 2021. These data will be crucial for the establishment of the real value of this ATP-competitive small molecule, alone or in combination with other drugs, for cancer treatment.

Literature data show the versatility of CX-4945 to synergistically cooperate with various chemotherapeutics directed against different targets such as DNA, kinases, structural components of the cytoskeleton and the proteasome, endowed with specific modes of action. For example, several of the studies mentioned in this review highlighted the efficacy of combining CX-4945 with different DNA-damaging agents [48, 51, 52, 55, 57, 58, 61]. Indeed, it is well-known that CK2 plays an essential role in preserving genome integrity by targeting XRCC1 and XRCC4, key players in DNA single- and double-strand break repair, as well as many other proteins involved at different levels in the fine-tuned process of DNA repair, such as MGMT, MDC1 and Rad51 [25]. Thus, a combination therapy based on a DNA-damaging agent and CX-4945, to hamper DNA-repair, may likely represent a feasible strategy to increase the vulnerability of tumour cells. Not less important is the contribution made by CX-4945 to overcome chemoresistance that cancer cells usually acquire after long-term drug exposure [25, 41, 47, 49].

Finally, it is to be mentioned that CX-4945 has recently been characterized as a methuosis inducer, a new issue that could be relevant in anti-cancer combination therapy. When used at high micromolar concentrations, ranging from 10 to 50 μM, CX-4945 promotes a distinctive form of cell death characterized by displacement of large, macro-pinocytic-derived and fluid-filled cytosolic vacuoles, that has been dubbed methuosis (from the Greek word μεθύω*,* to be drunk) [93–95]. We and others have previously reported that the induction of methuosis mediated by CX-4945, as well as its derivative CX-5011, is CK2-independent [93, 95]. Experimental data exonerate CK2 from being involved in the activation of macropinocytosis, since both the genetic reduction/ablation of CK2 subunits and the treatment of cells with kinase inhibitors structurally unrelated to CX-4945, failed to promote the aberrant formation of the cytosolic vesicles [93, 95]. Moreover, we showed that the ability of CX-4945 to promote methuotic cell death was cell type-specific since the extent of the vacuolization that followed CX-4945 stimulation considerably differed amongst the various cell lines used, distinguishing highly sensitive cells (i.e., HepG2 and GN11), moderately sensitive cells (i.e., MDA-MB-231 and HEK-293 T) and insensitive cells (i.e., HeLa). Although the concept of off-target effects has a negative connotation, the finding that CX-4945 promotes this non-canonical mechanism of cell death could represent an added value, rather than an obstacle, in the context of anti-cancer combination therapy for at least two reasons: 1) induction of methuosis could represent a feasible anti-tumour approach for those tumours that acquired the ability to escape from apoptosis or that developed resistance to classical apoptosis inducing agents; 2) enhancing macro-pinocytosis, CX-4945 could favour the uptake of additional drugs, thus representing an ideal condition for a pharmacological therapy that involves the use of multiple drugs, as shown in [41], where doxorubicin accumulation in tumour cells was greatly increased by CX-4945 co-administration, or as suggested by Colin et al. co-treating GBM cells with temozolomide and different methuotic-inducing compounds [96]. This latter effect would greatly favour a combination therapy, but future work will be necessary to exactly understand the effective contribution of methuosis induction in different types of cancer.

In conclusion, promising preclinical results have been obtained by combining CX-4945 with different therapies. Although the ability of CX-4945 to effectively promote anti-tumour outcomes by itself has not been fully defined yet, its ability to foster and sustain the action of other anti-neoplastic agents seems evident, thereby contributing to an orchestrated anti-tumour action. The ability to regulate multiple pathways turns this CK2 inhibitor into a versatile extra-weapon that may synergize with many different anti-cancer therapies.

## Abbreviations

ALL: Acute lymphoblastic leukaemia
AML: Acute myeloid leukaemia
CLL: Chronic lymphoblastic leukaemia
CML: Chronic myeloid leukaemia
DHFR: Dihydrofolate reductase
HNSCC: Head and neck squamous cell carcinoma
GBM: Glioblastoma
MGMT: O-6-methylguanine-DNA methyltransferase
NSCLC: Non-small cell lung carcinoma
